# “Chagas Express XXI”: A new ArtScience social technology for health and science education—A case study in Brazilian endemic areas of Chagas disease with an active search of chronic cases

**DOI:** 10.1371/journal.pntd.0009534

**Published:** 2021-07-21

**Authors:** Tania C. Araujo-Jorge, Roberto R. Ferreira, Rita C. M. Rocha, Thallyta M. Vieira, Nancy D. Costa, Luzia L. Santos, Josefa O. Silva, Marcelo O. Mendes, Juliana Almeida-Silva, Erik J. Costa, Rodrigo Mexas, Jonathan G. Oliveira, Ana M. Suarez-Fontes, Teresa C. M. Gonçalves, Catarina M. Lopes, Marcio L. Mello, Cristina X. A. Borges, Luciana R. Garzoni, Daniel Gibaldi, Joseli Lannes-Vieira, Marcos A. Vannier-Santos

**Affiliations:** 1 Laboratory of Innovations in Therapies, Education and Bioproducts, Oswaldo Cruz Institute (LITEB-IOC/Fiocruz), Oswaldo Cruz Foundation (Fiocruz), Rio de Janeiro Brazil; 2 Laboratory of Functional Genomics and Bioinformatics, Oswaldo Cruz Institute (LAGFB-IOC/Fiocruz), Oswaldo Cruz Foundation (Fiocruz), Rio de Janeiro, Brazil; 3 Center for Biological and Health Science, Universidade Estadual de Montes Claros, Minas Gerais, Brazil; 4 Rio Chagas Association, Rio de Janeiro, Brazil; 5 Institute for Science and Technology Information, (ICICT/Fiocruz), Oswaldo Cruz Foundation, Rio de Janeiro, Brazil; 6 Laboratory of Biology and Parasitology of Wild Reservoir Mammals, Oswaldo Cruz Institute (LABPMR-IOC/Fiocruz), Oswaldo Cruz Foundation, Rio de Janeiro, Brazil; 7 Interdisciplinary Laboratory in Entomological surveillance in Diptera and Hemiptera, Oswaldo Cruz Institute (LIVEDIH-IOC/Fiocruz), Oswaldo Cruz Foundation, Rio de Janeiro, Rio de Janeiro, Brazil; 8 Vice-Presidency of Environment, Healthcare and Health Promotion (VPAAPS/Fiocruz), Oswaldo Cruz Foundation, Rio de Janeiro, Brazil; 9 Laboratory of Biology of the Interactions, Oswaldo Cruz Institute (LBI-IOC/Fiocruz), Oswaldo Cruz Foundation, Rio de Janeiro, Brazil; Universidade Federal de Minas Gerais, BRAZIL

## Abstract

**Background:**

Chagas Disease (CD) affects 6–7 million people worldwide and is related to poverty-promoting conditions. Chronic asymptomatic cases are mostly invisible to health systems. Aiming (1) to translate CD discoveries into education/information practices to raise alertness and empowerment of affected people; and (2) to perform an active search of CD cases, articulating intersectoral actions to improve the access of infected people to the local health service for the treatment of CD; our research group developed and tested under field conditions as innovative social technology: an itinerant education interdisciplinary setting named “Chagas Express XXI” (CE21).

**Methodology:**

CE21 was created as an “imaginary train” with ~40 ArtScience workshops, games, laboratory activities and conversation circles. An entry/exit plus six activity modules combined associations of affected people, microscopic observations, One Health education, and wellness activities. CE21 was conceived as a social technology, since all the processes were co-created with CD patients and inter-sector local partners. Descriptive statistics showed quantitative data collected throughout the expeditions (CD knowledge, serological results). Qualitative data accessed the public perceptions about the education activities.

**Principal findings:**

CE21 was exhibited in local educational institutions (schools, universities) in four cities, engaging 2,117 people that evaluated the 41 activities carried out. Citizens and health professionals enjoyed acquisition of information related to blood, parasites, vectors, reservoirs, environmental changes, and social determinants of CD. Further, local legacies of 600 participants volunteer for health promotion groups and CD associations, local empowerment groups to fight for better health conditions, and 05 mural paintings. We observed that 81% of the participants ignored the possibility of treating CD while 52% of the participants requested a blood test for CD showing seropositivity in 20% of them.

**Conclusions:**

CE21 is a social technology potentially useful for health and science education and active search of asymptomatic CD chronic cases. Moreover, this technology may be adapted to understand and to cooperate in other potentially epidemic situations, especially NTDs related.

## Author summary

In the last decade one important issue concerning Chagas Disease (CD) is the active engagement of CD affected persons (patients, families, friends, students, and health professionals) as protagonists of a global struggle against neglection. Since 2015 CD patients of Fiocruz-Rio de Janeiro were in close contact with our education research group, where they were able to develop ArtScience activities related to information, education, and self-consciousness. This work paved the way for the creation of the Rio Chagas Association in 2016. However, its members often requested opportunities to offer activities of this nature in their birthplaces, where their families still live in a situation of socioeconomic vulnerability and at risk of infection. To address this request, our group developed a social technology called “Chagas Express XXI” (CE21), an expedition that offered 41 practical ArtScience activities that were co-created and co-mediated by people affected by DC, researchers, and graduate students. We tested CE21 in the Brazilian state of Minas Gerais, where 2,117 people filled out the participation forms. Among them, 1,100 adults requested the available serological test, and 20% were diagnosed with positive CD. These results support that CE21 could be used as a tool for active search of chronic asymptomatic cases. Further, 600 participants were registered to collaborate in health promotion nuclei to foster new associations of CD affected persons. Our study presents an innovative social technology to address prevention and health care for CD using non-formal learning and its corresponding educational resources. This education technology can be applied to other NTD by direct communication to people at risk of infection and/or affected in endemic areas. Science and health education are intimately related and the association with the art tools helps to improve knowledge communication to society.

## Introduction

Chagas Disease (CD), or American Trypanosomiasis, is a neglected tropical disease (NTD) [1] and affects 6–7 million people worldwide, leading to 12,000 deaths/year [2]. CD is caused by the flagellate protozoan parasite *Trypanosoma cruzi*, discovered in 1909 by Carlos Chagas [3]. Social inequalities strongly imply CD transmission and control because of its social determinants [4] leading to the recognition that the disease is a complex social issue [5,6].

The endemic nature of CD was unveiled in the middle of the 20th century [3], bringing a dramatic picture to Latin American countries, due to the great diversity of blood-sucking insects acting as vectors of *T*. *cruzi*. More than 100 species are already related to the transmission of *T*. *cruzi* after feeding on wild and/or domestic mammals that are natural hosts for the parasite [2]. Four main routes of infection sustain parasite transmission: (1) contact with feces and urine of infected triatomine in inadequate housing, work, and environmental conditions; (2) consumption of food or drink contaminated by *T*. *cruzi-*infected triatomine residues; (3) congenital route; and (4) blood transfusion and organ transplants. The acute infection lasts for 1–3 months, is mostly benign, unrecognized, and underdiagnosed. The infection persists for an asymptomatic chronic indeterminate period that can last a lifetime in most people. During this time, about 70% of the infected people are unaware of the infection and it is estimated that only 10% of cases are adequately diagnosed by laboratory tests and that less than 1% are properly and timely treated with available trypanosomicide drugs [7]. The remaining, about 30% of chronically *T*. *cruzi*-infected persons, evolve with relevant health problems such as cardiopathy that may progress to heart failure and digestive complications [8]. The global costs related to CD are estimated at US$7.19 billion per year, with a substantial proportion of the burden resulting from productivity lost from cardiovascular disease-induced morbidity and early mortality [9]. After years of discussion, a Therapeutic Guideline for CD Diagnosis and Treatment–PCDT Chagas–was elaborated by the Brazilian Ministry of Health in 2018 [10], and although CD still affects millions of people, municipalities face enormous challenges in implementing the new PCDT. According to the Pan American Health Organization [2], CD is a compulsory notification infection in the acute phase in all endemic countries. In response to social movements and also to scientific advice, the Ministry of Health recently decided to make notification of chronic CD also mandatory [11]. Brazil is the first country in the world to adopt this normative, which represents a great achievement for the movement led by the International Federation of Associations of People Affected by Chagas Disease (FINDECHAGAS) and other non-governmental organizations [6].

Despite the amount of biomedical information on CD [2,6], it is mostly invisible to society and education is an issue stressed as necessary since the centennial of CD discovery [12]. People from endemic areas are generally not aware about the risks related to transmission nor about the implication of taking the proper medicine as soon as they are diagnosed [7,13,14]. The social debt with CD affected people needs to be redeemed with a robust partnership between public entities and the organized civil society. The visibility to these NTD trends increases both through websites and Internet video channels of international organizations, such as the World Health Organizations (WHO), Oswaldo Cruz Foundation (Fiocruz), FINDECHAGAS, Chagas Coalition and others.

The location and visibility of chronic patients is still a challenge of the new compulsory chronic notification policy in Brazil, in addition to guaranteeing the right and integral and healthy care of these patients. However, if CD vulnerable persons do not know the risks or how they can be infected, they could not care about its consequences for their own health or the health of their families. The stigma of having CD is psychologically heavy, representing a social burden for those affected [15]. Some patients refuse to share the diagnosis with their relatives, while others become depressed and may even commit suicide [15]. These multifaceted situations can be faced with the best “vaccine” against fear, which is knowledge and dialogue, as proposed by Paulo Freire [16]. This is what the “Chagas Express XXI” (CE21) is expected to bring to the endemic areas where CD vectors are still a risk for *T*. *cruzi* transmission and deserve continuous environmental and health surveillance where both health professionals and citizens are not well informed, communicated nor educated concerning threats related to both the acute and chronic CD [6,7,13].

### ArtScience as a strategy for health education and active search of chronic cases of Chagas disease

ArtScience is a growing area in which artists and scientists collaborate to foster creativity and face complex problems [17,18]. As a strategy for education, ArtScience is under intense studies in research fields as health [19], management [20], and teaching [21], commonly refereed as STEAM (science, technology, engineering, arts, and mathematics). STEAM is an acronym used to propose innovative and interdisciplinary educational curricula and has been spreading in many countries in the last two decades when science education encounters the arts [22–23]. ArtScience offers a conceptual advantage concerning more conventional education alternatives due to inter and transdisciplinary approaches that favor health innovation in education [19]. ArtScience is an explicit neologism for the fusion of both fields, formally proposed in the 2011 ArtScience Manifesto [17]. The Manifesto proposes 17 theses, starting with three assumptions that paved CE21 ideas: (1) Everything can be understood through art but that understanding is incomplete; (2) Everything can be understood through science but that understanding is incomplete; (3) ArtScience enables us to achieve a more complete and universal understanding of the phenomena. The sixth thesis of the ArtScience Manifesto states that: (6) ArtScience is not embodied in its products so much as it is expressed through the convergence of artistic and scientific processes and skills [17].

In Brazil, after a long experience mixing science, education and art, we adopted the term “CienciArte” (in Portuguese, ArtScience in English) for inter and transdisciplinary research, workshops and courses that develop ArtScience approaches [24]. In addition, we apply Paulo Freire’s concepts of dialogue, joy and questioning of reality, which means direct interaction with people to foster autonomy and political awareness of the causes and implications of a given social problem [16,24]. We also worked with educational activities to tackle many health subjects, including NTD as CD and arboviruses [19,25]. This effort converged with the urgent need for formal and non-formal education strategies, materials, resources, and technologies concerning NTD, reinforced in 2017 by the WHO initiative in creating a Technical Group on Information, Education and Communication to control CD [6].

From 2015 to 2018, CD patients followed up at Fiocruz/ Rio de Janeiro were invited to participate in ArtScience workshops in a course entitled “Talking about Chagas with ArtScience” [25], an education initiative that paved the way for the creation of a civil society organization that congregates people afflicted and affected by CD (“Rio Chagas Association”). Since 2018, these Rio Chagas members requested Fiocruz researchers to deliver health contents to people living in areas where they came from, and where their families still live, mostly in socioeconomic vulnerable situations as in Minas Gerais, Pernambuco, and Paraiba, all states of Brazil. In response to this challenge, those Rio Chagas members together with a group of researchers and Master and Doctorate students from Biosciences and Health Education Post Graduation Program, developed the itinerant ArtScience expedition named “Chagas Express XXI” (English acronym: CE21; “Expresso Chagas XXI”, in Portuguese).

The objectives of the present research study were: (1) to translate CD discoveries into education/information practices leading to raise awareness and empowerment of affected people through the conception, development, and testing in field areas a non-formal social educational technology (CE21) to present and discuss CD issues. Also (2) to perform an active search of CD cases, by associating intersectoral actions to improve the access of infected people to the local health service for treating CD and CE21 testing as a strategic tool for active search of chronic asymptomatic CD cases. The connection between these objectives is based on the translation of CD science knowledge and discoveries into education/ information procedures and practices (objective 1) in a socially attractive technology (CE21) that could help to perform active search of chronic CD seropositive cases (objective 2). Therefore, raising interest in the theme and awareness of the population, and affected people would empower them to fight for their right to specific CD diagnosis, treatment, and care in the public health system.

## Methods

### Ethics statement

The project and all the consent forms and questionaries were previously analyzed and approved by the Ethical Committee of Research in Humans of the Oswaldo Cruz Institute (CEP-IOC/Fiocruz, CAAE 15584119.4.0000.5248) according to Brazilian laws and regulation of research with humans. During the expedition, all the participants were identified by filling a registration form for authorization of audio and video image use for communication and research items, as well as some objective questions regarding knowledge about CD that will be presented in Tables organized in the Results section. A formal written consent was obtained from each participant or from his/her parent/guardian in case of under the age of 18. The individuals pictured in Figs 2, 3 and 5 have provided written informed consent to publish their image alongside the manuscript. The authors own the copyright of the images and the poster templates, since the art designer is EJC, one of the co-authors.

### Theoretical-methodological basis for the Chagas Express XXI conception

This study uses the theoretical-methodological background of ArtScience in a transdisciplinary approach for innovation, as proposed by the ArtScience Manifesto [17] and by the ArtScience approach presented by Todd Siler [26,27]. “Imagine, connect, and discover” are the first three steps of the ArtScience approach, and also the foundation for basic research in all science and humanity fields [19,24,25]; “invent, apply, and innovate” are the last three steps. Discoveries continuously feed the scientific literature but only when translated into products, processes, and tools they can turn into innovations, causing an impact of various nature in the social context; adding either tangible or intangible value [19,24]. All CE21 activities were founded in the 13 thinking tools / cognitive categories, described by Robert and Michèle Root-Bernstein [28], and intensively used in our ArtScience education workshops [19,24], as to know: (1) Observing and registering, not simply watching, to go beyond the visual aspect of seeing; (2) Imaging, evoking images, creating visual representations in the mind; (3) Abstracting to take something and to simplify it to its most important single element, to imagine what something could be that it is not really is; (4) Recognizing patterns, identifying what is common and what is unique; (5) Forming patterns, creating something different by combining two or more elements together; (6) Making analogies, finding a relationship in size, function, form, or other; (7) Thinking with the whole body, moving the body through space to let imagination flow; (8) Empathizing, putting oneself in someone else’s position, changing the perspective and the point of view; (9) Thinking in a dimensional way, moving from 2D to 3D, 4D (including time, movement and sensorial inputs), or 5D (including symbolic representations), scaling, or altering the proportions and symbols; (10) Modeling, creating representation of something in a physical (and even functional) form; (11) Playing, simply for the fun and for the joy of doing something; (12) Transforming, altering some thing or some tool into another thing or another tool; (13) Synthesizing, describing a complex and whole idea in few words, in a picture, or in a movement or sound. These categories are important to promote and to consolidate creativity [24–26]. Besides, they also help participants to think and cross the imagination barrier, making it possible to reflect and raise awareness about the issue in question. In CE21 art is not a utility, a tool, but a method, a language, a skill to be presented, to be learned and to be appropriated by the participants. Art does not bring beauty or aesthetic, like a cherry in a cake (even when it adds beauty). Art opens a way to perceive nature and environmental problems from a new perspective, trying to generate challenging questions that lead the participants to think and to talk about CD problematics. We then purposefully mixed dialogical and interactive activities with other conventional and/or direct information activities, always using the ArtScience approach.

### Artistic conception

**Fig 1:**
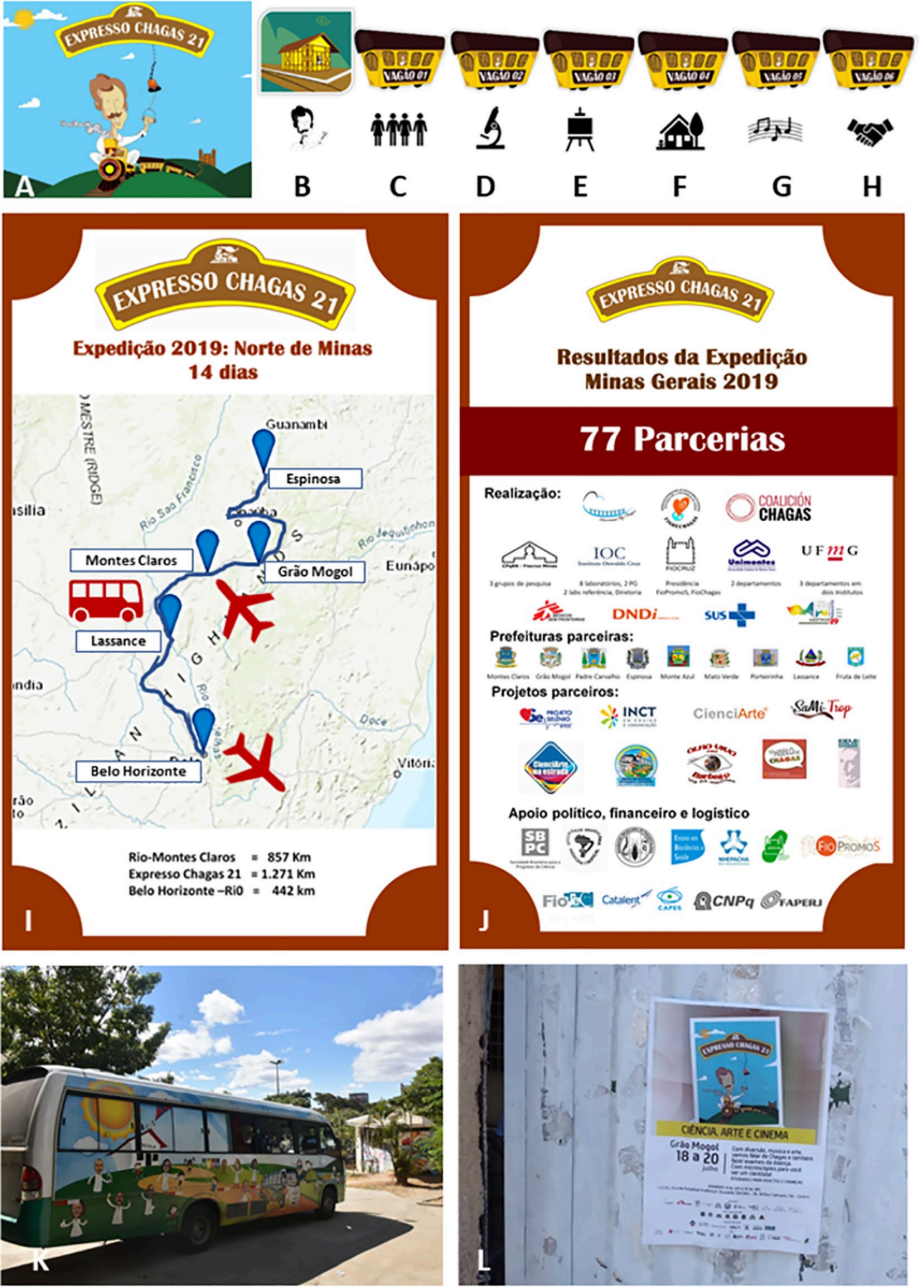
Materials and images of the Chagas Express XXI expedition to the northeast of Minas Gerais, Brazil, in winter vacation, July 2019. Art concept of Chagas Express XXI created by Erik J. Costa (A—J). The main pamphlet (A), the entry station (B) the successive wagons (C,D,E,F,G,H) and the general template of the banners (I,J) prepared as part of the exhibition composing expedition; the travel route and vehicles (prepared over a map that was in public domain - https://store.usgs.gov/map-locator) (I) and partnership logos of the projects network, cooperating institutions and municipalities in the presentation of activities (J); mobile laboratory in the “Science on the Road” project bus that merged the team (K); local poster to publicize scheduled activities (L).

A Master’s student at Fiocruz, who teaches art at an elementary school art, and co-author of this manuscript (EJC) idealized the artistic concept of CE21 presented in Fig 1. The main CE21 tool was in the format of an “express train” driven by a caricature of Carlos Chagas (CD discover, Fig 1A), arriving at a scenography “train station” (Lassance station). The entrance and the exit (Fig 1B) were also represented, followed by a set of six “wagons” / education modules (Fig 1C–1H) comprising the imaginary train with various recreational activities.

### Data collection and analysis

The combination of qualitative and quantitative methods [29] is a trend in research on science and health education [30]. Inspired by previous expeditions aimed at addressing poverty-related NTD problems [31], with no records from similar experiences, this is a case study in which triangulation [32] helped to achieve reliability and validity. Data triangulation (using multiple data sources to help understand CE21) and triangulation of methods (using various research methods to study CE21 combined with qualitative and quantitative methods for a reliable description of the developed social technology) were used along with investigator triangulation (using multiple researchers to collect and interpret data).

This study was designed using two qualitative methods of data collection [32]: (i) documentary analysis [33,34] to gather and classify all the project documents to generate a precise description of this social technology; and (ii) autoethnography [35,36] and ethnography to gather the perceptions of the authors embedded in the expeditions and also of the participants that recorded oral testimonials. As quantitative methods, we used: (iii) questionnaires filled at the entry station aimed at describing the socio-geographic profile of the participants as well as their basic knowledge and major gaps about CD; (iv) Emoji Likert-scale evaluation forms [37] to semi-quantify participants´ opinion about each major set of activities; and (v) serological profile of the participants (CD positivity).The documentary analysis allowed researchers other than the CE21 team or authors to access the social technology documents for close investigation aiming at the characterization of CE21 as a social technology [38,39]. As stated by Shaw et al (2004) “*documents may be the only source of data at an early stage of a policy innovation*” [33]. Open documents were retrieved with keywords “Expresso Chagas” in two public open data platforms: (i) the Brazilian Ethical Platform for research projects with human participation (http://plataformabrasil.saude.gov.br) and; (ii) the Oswaldo Cruz Foundation repository (www.arca.fiocruz.br), with special attention to posters used in the expeditions (www.arca.fiocruz.br/handle/icict/41554) and to inviting letters to municipality stakeholders, technical reports to funding agencies and media clipping.

**Fig 2:**
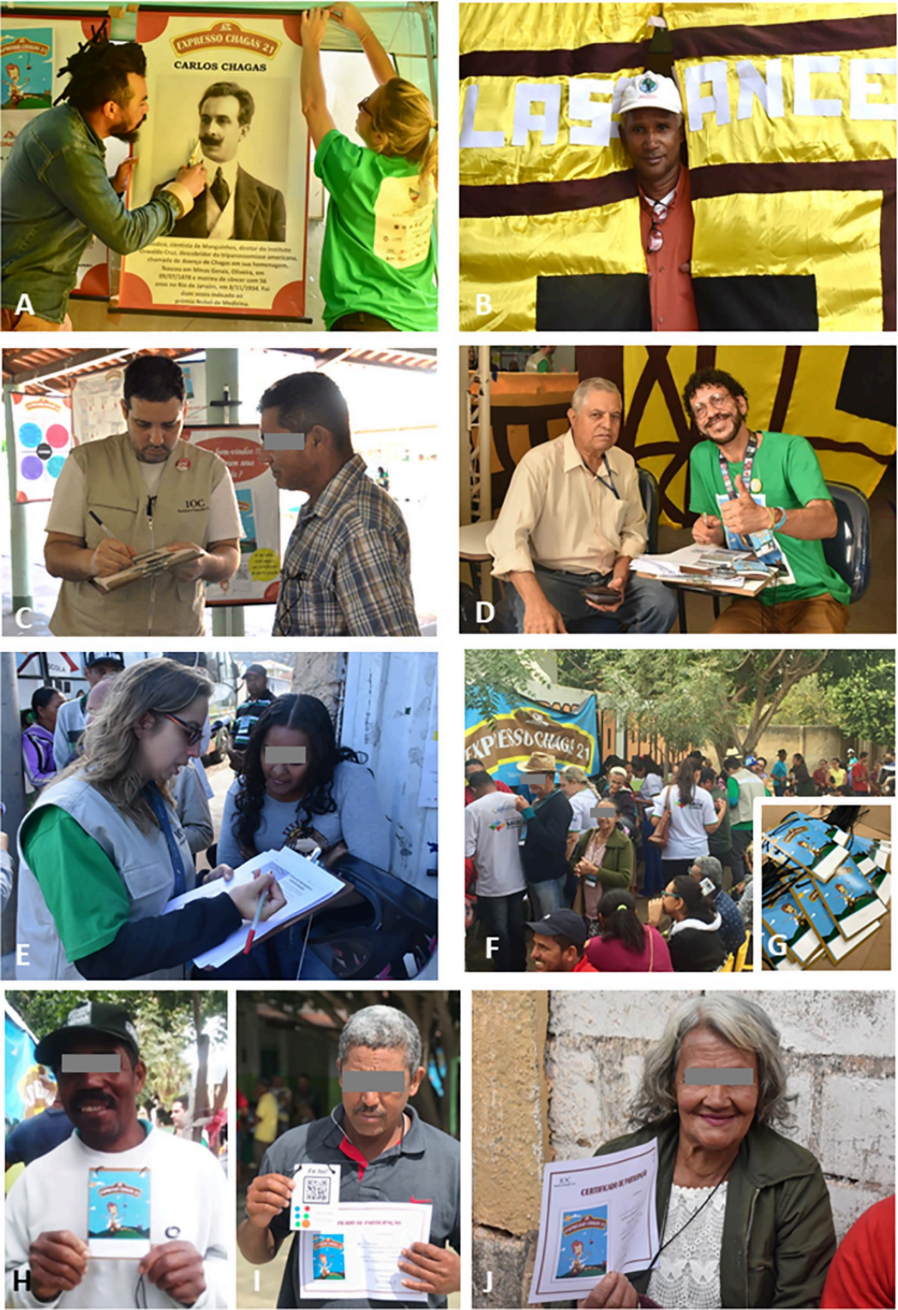
**Images of the Station entry and exit points:** (A) Setting up the exhibition and presenting Carlos Chagas, the scientist who discovered the disease in 1909; (B) Scenography of the exhibition entry named station “Lassance”, alluding to the village where the discovery took place; (C,D,E) Filling forms for each visitant; (F) crowded waiting line to attend the activities in a local school at Espinosa city, Minas Gerais; (G) a set of badges (identification cards) for the visiting public. Note the green T-shirt that identified the field team of the project (A,D,E) and the field jacket (C) that is used in field institutional missions (C,D); (H,J) proud visitants showing their front (H) and back (I) views of their badge and certificate (J) smiling to express joy and happiness.

Since all authors participated in CE21 conception and implementation, we used autoethnographic and ethnographic approaches and collected testimonials as well as mail impressions from both the participants and the CE21 research team. This set of information generated a rich amount of qualitative data concerning perceptions, remarks, discussions expressed in oral testimonials that emerged before, during, and after each day in the expeditions. Images, videos, texts, and audio pieces were used as sources of qualitative data and perceptions. The CE21 complete working force comprised 57 persons (enrolling about 30–40 persons in each city) distributed as: (a) 07 CD affected persons and members of the Rio Chagas Association, (b) 21 researchers associated to Fiocruz Institutes in Rio de Janeiro (12 researchers/post docs) and Belo Horizonte (04 researchers), as well as to the State University of Montes Claros—Unimontes (01 professor), to the Federal University of Minas Gerais (01 professor), and to the SaMi-Trop project in Minas Gerais (03 researchers), (c) 25 students from Rio and Minas Gerais universities (08 PhD, 06 MSc, 06 undergraduate, 03 specialization and 02 high school students), (d) 04 supporting staff (2 drivers, 1 photographer and 1 laboratory technician). The students were invited directly by their research supervisors or after attending a training course on ArtScience previously carried out to test all the education activities proposed and formatted for the expedition. All the CE21 team members (Fig 2A) wore project T-shirts, jackets, backpacks, and badges to identify their positions and functions in the work groups. They also carried cell phones and field notepads for personal field notes. Daily recorded meetings occurred during field activities to report collectively on the experiences, problems, and successes. Preparatory workshops and courses also occurred prior to the expedition, as well as review and assessment crew meetings at the end of field experiences.

For quantitative data collection, all the participants were asked to answer a questionnaire (both virtual and in printed sheets, depending on the local internet conditions, Fig 2C–2E) at the entry of the exhibition (module 1: Lassance station, Fig 2B) and at any time in complementary surveys at the different exhibition modules (wagons 1 to 6). The frequencies of each type of answer were calculated and, when it was the case, were compared using Chi-square analysis to indicate significant differences when p<0.05. At the end of the exhibition, the participants were invited to evaluate each block of activities in a quali-quantitative approach using a Likert five levels chart [36] using emoji: (1) I hated it; (2) I didn´t like it; (3) I liked it more or less; (4) I liked it very much; (5) I love it. Free opinions were also recorded, both by voice recording and by notes taken by the research team in their field notebooks. All participants received visiting badges with personal identifications and filled the proper consent forms previously approved by the Fiocruz Ethics Committee.

### Study area, expedition roadmap and epidemiological and serological study for active search of Chagas disease chronic cases

The criteria for choosing cities to visit in the field expedition was related to (i) the number of people with CD (the estimated prevalence) as informed by the Health Ministry (federal level) and Secretaries (state and municipality levels); (ii) family and affective ties that members of the Rio Chagas Association held within the proposed cities (birth place, family and friends living in that area, links with community leaders and stakeholders), and (iii) the engagement of the respective communities in receiving the expedition, thus preparing a counterpart of preliminary activities and local organization. The counterpart of the municipality included (a) the choice and assignment of a space from a public school or university area suitable to receive the exhibition free of charge for the project and which could also participate by providing education staff as a complementary team (this condition varied in dimension and in engagement according to each city visited), (b) health personnel related to blood sample collection, management and storage, as well as laboratory infrastructure / partnerships for future CD diagnosis and notification, (c) mayor commitment for providing future healthcare for positive CD cases found actively.

The final roadmap (Fig 1I) showed the four cities to be visited by the expedition (a) Grão Mogol (GM, 15,667 inhabitants; 1.9% estimated CD prevalence); (b) Espinosa (Esp, 31,624 inhabitants; 0.7% estimated CD prevalence); (c) Montes Claros (MOC, 404,804 inhabitants; 0.7% estimated CD prevalence) and (d) Lassance (Las, 6,490 inhabitants; 0.5% estimated CD prevalence). Number of inhabitants were obtained from the Brazilian population census (https://censo2010.ibge.gov.br/) and CD prevalence was calculated from the Brazilian Ministry of Primary Care Health System data (http://tabnet.datasus.gov.br/cgi/deftohtm.exe?siab/cnv/SIABSBR.DEF). GM and Las were the birthplaces of two Rio Chagas members (co-authors in this paper, NDC, LLS) who are currently on the board of “Rio Chagas” CD Association. Lassance was the historical town where Carlos Chagas first noticed a domiciliated triatomine vector and described *T*. *cruzi* in the blood of domestic animals and in the febrile child that originated the first description of Chagas disease [3]. Esp and MOC were cities suggested by the Minas Gerais State Health Service as important sites where CD could be reemerging, as reported by local epidemiological data. In GM, Esp and Las the exhibition was planned to be carried out in public schools. MOC was the largest city in the region and the university professors were the main partners, leading the health, education, and communication sectors, thus offering the Health Center Building central hall as the exhibition area and inviting actively the local health community agents to attend the exhibition.

At the end of the expedition, a stop in Belo Horizonte (BH) the capital of Minas Gerais state was planned to present CE21 to the academic community attending the annual meeting of the Brazilian Societies of Tropical Medicine and of Parasitology (MedTrop), at the Federal University of Minas Gerais. BH profile of participants was of undergraduates, researchers, and CD experts added to topics in the fields of Parasitology and Tropical Medicine since the city has not registered active transmission by triatomines (BH was not considered as an endemic area).

In three cities, (GM, Esp and MOC) serodiagnosis was offered to participants who were interested in knowing whether they could have been infected with *T*. *cruzi* and thus be eligible for treatment and care for CD. This procedure was not designed as a seroepidemiological survey for incidence nor for prevalence data, since it relied on the personal expectation of the participants to get to know their own health condition concerning CD status. After visiting the first two modules (wagons 1 and 2) the participants were asked if they were interested in collecting blood for a serodiagnosis that would be reported after 30–60 days at their own primary healthcare unit. For all those who decided to test for CD, a password number was provided in wagon 2, specific questionary forms were further filled with the proper information requested in the local health services. Blood collection was carried out in a safe field laboratory environment and involved the local health team, who helped the CE21 team. Blood was further processed for serum collection, frozen at -20°C and stored at -80°C at Unimontes. The identified samples were then sent to (1) the Brazilian National Reference Center for CD diagnosis (Ezequiel Dias Foundation—FUNED, Belo Horizonte, Minas Gerais*)*, that used ELISA and an indirect hemagglutination assay (IHA) for all the samples collected at GM and MOC. Samples were also sent to (2) the São Paulo University Blood Center (USP) responsible for SaMi-Trop study (a partner of CE21), that used two serologic assays: a *T*. *cruzi-*lysate-based enzyme immunoassay and a recombinant enzyme immunoassay [40]. According to the PCDT recommendation [10], the final diagnosis was confirmed only for persons with two positive serologic tests against *T*. *cruzi*.

## Results

### Chagas Express XXI description: the context and history of an ArtScience expedition and a social technology for non-formal science and health education

Documentary analysis on plans, forms, questionaries, banners, posters, virtual educational materials, photo-books, image records made by participants in different workshops, and quantitative datasheet allowed the following CE21 description:

*Conception*: CE21 was developed and co-created by researchers and students at Fiocruz and by CD activists who participate at Rio Chagas Association (www.facebook.com/Riochagas.2016/). This is the main reason to consider CE21 as a social education technology for health promotion and for the active search of CD carriers at the local level. The concept of social technology was revisited in Brazil [38,39] and is defined as: (i) a solution developed in a partnership between the academy and the sectors of civil society, (ii) in a shared way, and (iii) adding value to society, whether monetary or intangible (cultural, quality of life, among others). CD affected members at Rio Chagas Association profoundly inspired CE21 proposals thus interfering deeply in its conception and development. An example was the project logo, which was initially designed with the Roman numerals “XXI”, denoting CD challenges in the twenty-first century. Some CD affected persons did not identify nor recognize Roman numerals and wondered about the meaning of the “XXI”. This was an opportunity for them to learn about historical facts and the proper way to cite the centuries but also a lesson for the researchers to propose more identifiable communication symbols to the local persons. This experience led us to decide to change the logo to the Arabic numerals “21” (Fig 1A,1I and 1J).

*Aims*: CE21 proposes interactive and ludic ArtScience activities about CD related issues in regions where people face the chronic disease (large cities and ancient migrant rural families and small country cities in triatomine endemic areas). In addition, where contamination by the parasite through any route causing the disease is a risk, according to the last national panorama reported by governmental authorities [11]. The activities (banners, microscope and lenses experiments, games, mini-workshops, minicourses, and conversation/ participation circles) were organized in an exhibition, in the form of an imaginary train, alluding to the train car adapted as the doctor’s office and laboratory room where Carlos Chagas discovered the CD causing parasite, *T*. *cruzi*. In 1909, the parasite was related to the tropical American Trypanosomiasis disease, also described by Chagas [3]. The technology aims to: (1) promote health and education with joy; (2) stimulate the creation of new associations of CD affected people, extending their voices and visibility; (3) publicize the new Brazilian Clinical Protocol and Therapeutic Guidelines for Chagas disease [10], encourage access to diagnosis; (4) revisit Chagas’ discovery with citizens of the visited area; (5) retake the campaign for treatment and disseminate therapeutic innovations; (6) bring hope for chronic CD affected people, based on their own testimonials and on dissemination of innovations to face the disease and (7) facilitate active search for chronic cases referring to primary care and support the organization of local lines of care.

*Logistic*: Agreements were previously established with local stakeholders related to health, culture, and education sectors to enable the exhibition in four different cities (Fig 1I), thus defining an inter-sector action and providing CE21 installation in local school areas. In addition, the invitation to health professionals to visit the exhibition and participate in the activities, to learn scientific and technical contents were made possible. For logistics, as well as for funding the expedition, broad partnerships were settled (Fig 1J). All funding for travel expenses (57 persons) were covered including expenses with the “Science on the Road” bus (Fig 1K) sent to fieldwork from Rio de Janeiro to Minas Gerais (857 km) carrying various materials and equipment previously assembled (Fig 1L). Contact with local media to casting information on the activities´ places, dates, and time schedules were also cared for. This was the municipality counterpart agreed to host CE21 expedition. The co-authors of this paper as well as all the persons acknowledged and cited nominally comprised the team.

*Exhibition content*: the expedition roadmap (Fig 1I) was exposed at the entrance of the schools or universities where the exhibition was held. A set of 55 posters and 3 banners was used to present contents or to visually call the attention of the public for the proposed interactive activities (Fig 1J). All the posters and banners are available at: www.arca.fiocruz.br/handle/icict/41554. The “Science on the road” laboratory bus project (Fig 1K) [41] and some flyer communications (Fig 1L) integrated the set of art materials used in the expedition. The banners set up (Fig 2A) and the scenography setting (Fig 2B) of the exhibition in schools or universities, conjugated with the previous call of the interested population (Figs 1L and 2C) prepared the stage for all the activities offered (Table 1). The first poster posed the following question to the participants: “Did you know that Chagas disease was discovered in Minas Gerais, in a train wagon?”. This poster showed pictures (1) taken in the Lassance station in 1909 and 2019, (2) of Carlos Chagas examining a child, (3) of pre-historic findings of triatomine rupestrian pictures and (4) of mummies from where *T*. *cruzi* DNA was extracted. These images helped to introduce the historical narrative that Carlos Chagas (Fig 2A) lived and worked in an adapted train wagon at the Lassance station to carry out studies on malaria between 1907 and 1909 [3] when he noted a novel microorganism in the blood smears of local animals and people. His observation led him to discover a new parasite and to name it *T*. *cruzi*, after his adviser, Oswaldo Cruz [3]. The participants were then invited to remake Chagas discovery by carrying out the CE21 activities.

**Table 1:** Chagas Express XXI ArtScience activities.

# Area	Activity
1. Station	1. “Your selfie with Carlos Chagas”: reception and accreditation
2. Station	2. “What word comes to your mind when you think about Chagas”: mini-workshop
3. Station	3. Short videos with Chagas disease affected persons´ reports
4. Station	4. “Conversation with the Trypanosome”: theater sketch on the waiting line
5. Station	5. Soundtrack: a) Life can be better- Luna Cohen; b) Health- Rita Lee
6. Wagon 1	1. Associations of Chagas disease affected persons (from Brazil and other countries)
7. Wagon 1	2. Support Associations pages and our social medias: Instagram @expressochagas / Facebook: Expressochagas / YouTube: Expresso Chagas
8. Wagon 1	3. “The History of Rio Chagas”: association from Rio de Janeiro
9. Wagon 1	4. “Our memory of Chagas”: memory game displaying images associated to histories of Chagas disease affected persons.
10. Wagon 1	5. “Your look”: photography mini-workshops (with passwords)
11. Wagon 1	6. Album of portraits, news, and cartoons
12. Wagon 1	7. “Craft workshop”: scented sachets
13. Wagon 1	8. “How to build a local Chagas disease Association?”: meetings with interested participants
14. Wagon 2	1. “Redoing Carlos Chagas discovery”: eyes at the microscope and magnifying glasses to discover *T*. *cruzi* and kissing bugs
15.Wagon 2	2. Medicines to treat Chagas disease
16. Wagon 2	3. New CD treatment protocol (PCDT) and comprehensive care
17. Wagon 2	4. “FluorArt”: mini workshop
18. Wagon 2	5. Testing for Chagas disease (with passwords): blood collection
19. Wagon 2	6. Innovations on the way: selenium treatment
20. Wagon 3 21. Wagon 3 22. Wagon 3 23. Wagon 3 24. Wagon 3 25. Wagon 3	1. “Portinari & Health”: mini-art education workshops 1.1. Reinterpretations of Portinari’s artworks, master pieces 1.2. Free drawing and living model 1.3. Biopsychosocial determinants of Health in Portinari´s artworks 1.4. Toys and games construction with recycled materials 1.5. Music evocated from re-readings Portinari´s artworks
26. Wagon 3	2.1. “Giant artery”: entering a giant artery to search for the parasite in blood
27. Wagon 3	2.2. “Modeling blood elements you saw”: mini-workshop
28. Wagon 4	1. “What are the kissing bugs in Minas and Brazil?”: mini-workshop
29. Wagon 4	2. “Biodiversit´Art”: mini-workshop
30. Wagon 4	3. “How to avoid kissing bugs”: mini-workshop
31. Wagon 4	4. “Visiting Virginia Schall´s House”: hunting for CD vectors and other dangerous bugs (an interactive house)
32. Wagon 4	5. “Infecting”: a game to play
33. Wagon 4	6. “Hunting for kissing bugs and getting to know the triatomine interactive points (TIPs) in the region”: a game to play
34. Wagon 4	7. “Hand washing and biosafety”: a game to play
35. Wagon 5	1. Aromas and health with self-massage
36. Wagon 5	2. “Music is health” group workshop
37. Wagon 5	3. Physical exercises that promote health
38. Wagon 6	1. Welcoming impressions and stories
39. Wagon 6	2. Participating in the ongoing research project
40. Wagon 6	3. Evaluating Chagas Express XXI with bullets
41. Wagon 6	4. Your photo on the Chagas Express collective panel

### Proposed participant flow and rational for presenting Chagas Express contents

**Fig 3:**
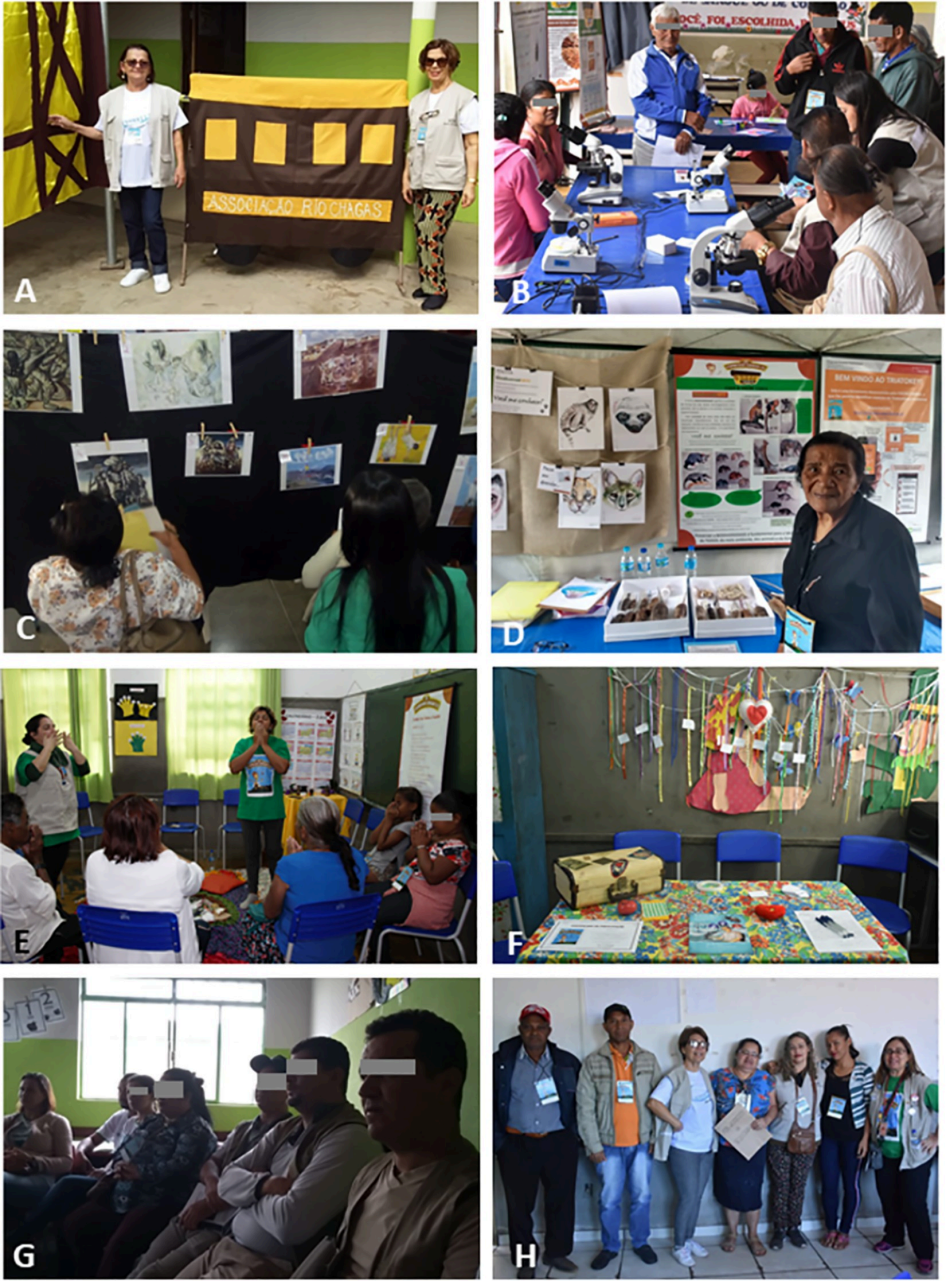
**Images from each of the six wagons showing some thematic activities and strategic meetings**: (A) wagon 1 with two members of Chagas disease from Rio Chagas Association that follow healthcare in Fiocruz (Rio de Janeiro); (B) wagon 2 with public observing parasites and insects under optical equipment; (C) wagon 3: re-readings of Portinari´s artworks; (D) wagon 4: Biodiversit´Art activities showing animal reservoirs; (E) wagon 5: self-massage workshop with adults and children; (F) wagon 6: craft drapery pending the most important perceptions written or spoken by the participants; (G) mini-courses for health professionals to present the new clinical protocol and therapeutic guidelines for Chagas disease (PCDT-Chagas); (H) compromising participants to organize new associations of CD affected persons, in this case at the city of Grão Mogol during the activities of wagon 1 of Chagas Express XXI in July 2019.

**Fig 4:**
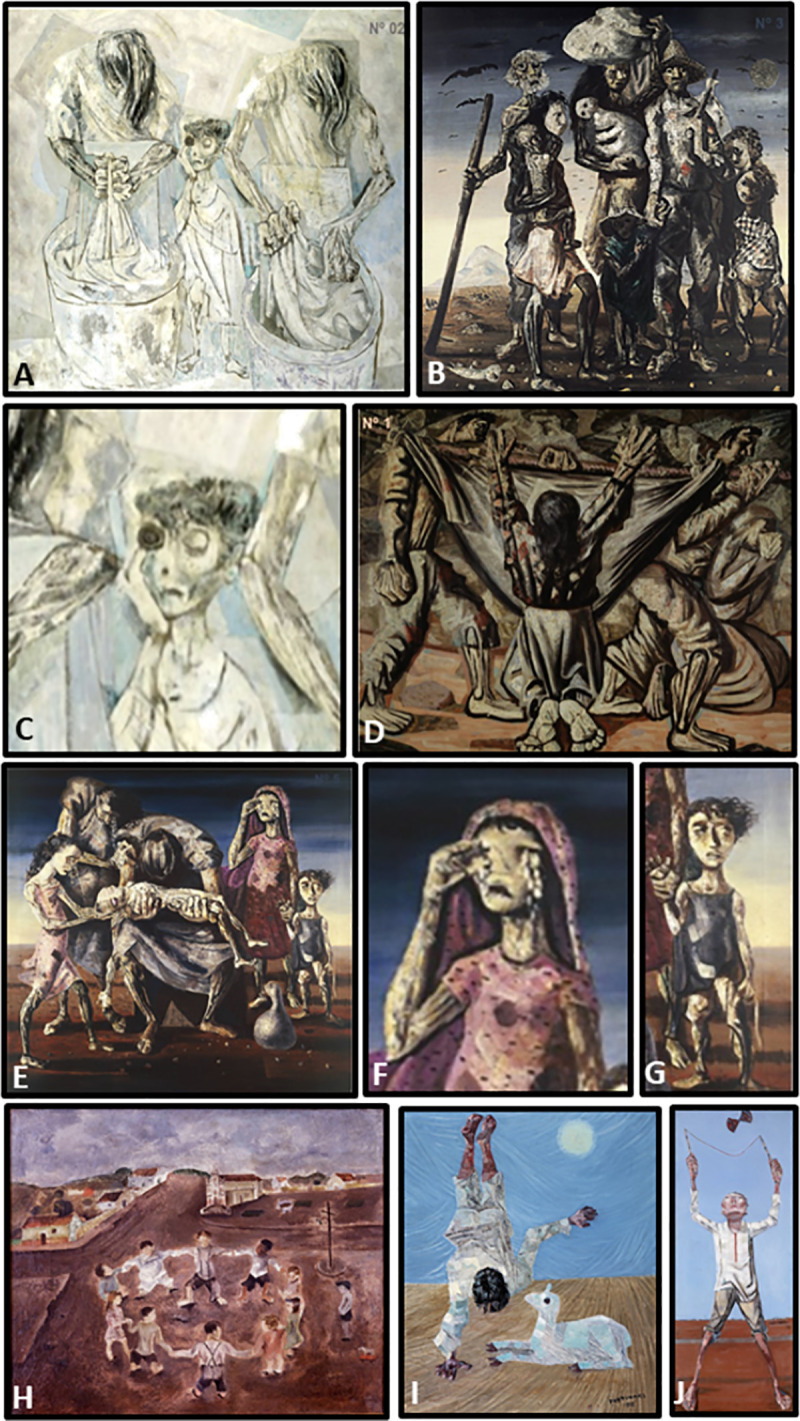
**Images of some paintings of Candido Portinari presented to the participants in wagon 3:** (A) “*Washerwomen*”, painted in 1944, size 170 x 200 cm; (B) “*Retreatants*”, painted in 1944, size 190 x 180 cm; (C) “*Washerwomen*”- detail of the central character with Romaña´s sign in the left eye; (D) “*Burial in the hammock*”, painted in 1944, size 180 x 220 cm; (E) “*Dead Child*”, painted in 1944, size 180 x 190 cm; (F) “*Dead Child*”–detail of woman with falling tears; (G) “*Dead Child*”–detail of a child character with Romaña´s sign in the right eye; (H) “*Children circle*”, painted in 1932, size 39 x 47 cm; (I) “*Upside down*”, painted in 1956, size 55 x 46 cm; (J) “*Diabolo*” (a game in which a two-headed top is thrown up and caught with a string stretched between two sticks), painted in 1959, size 154 X 75 cm. Their technical characteristics and museum locations are found in www.portinari.org.br.

Identified at the scenography Lassance station (Fig 2C–2E), the participants answered questions concerning demographic profile and general knowledge about CD (see below), afterwards, they were welcomed to explore all the exhibition modules and follow throughout the thematic “wagons” (Figs 1B–1H and 3A–3F), described as follows: (1) ASSOCIATIONS (Fig 3A): to know the Federation of CD organizations (FindeChagas) and its affiliated associations (in our study, Rio Chagas), their endeavor and organization; (2) LABORATORY & INNOVATIONS (Fig 3B): to know the instruments for the diagnosis and treatment of CD and to get to know the parasite and vector using, respectively, the microscope and magnifying glasses, as well as art activities designed for observation of parasites and cells; (3) DISCOVERIES & PLAY (Fig 3C): to enter a portable giant blood vessel inspired in a previously conceived giant model [42], and to observe Portinari´s art pieces (Fig 4A–4J) related to discovery of biopsychosocial determinants of the disease and to play with different toys handcrafted from recycled materials; (4) HOME & ENVIRONMENT (Fig 3D): to learn about the anthropization gradient and conservation process in the maintenance of wild cycle of transmission, risks in different environments, the diversity of transmitting insects and reservoir mammals taking the necessary attention at home and around surroundings in a One Health concept [43], thinking through art about the socio-environmental determinants of the disease; (5) WELL-BEING (Fig 3E): to exercise self-care with self-massage, music, dance, aromatherapy and other integrative health practices; (6) YOUR VOICE (Fig 3F): to interact with the team, declaring their opinions about the experience at CE21 and get involved in research projects, after signing specific terms of consent. Table 1 shows the themes presented at each of these spaces. Wagon 3 (Discoveries and Play) was the most original one since we do not know any similar initiative in CD education/ social literature. Paintings of the Brazilian artist Candido Portinari (Fig 4A–4J) freely accessible for educational purposes were chosen to promote reflections and dialogues related to some social determinants of health (briefly shown in Fig 3C), such as quality of life including poverty, nutrition, and housing, and to trigger infancy memories in the adult participants. Images of those paintings can be browsed at the Portinari virtual art gallery (www.portinari.org.br). On this website, it is possible to search for image content using keywords. The artist produced a series with iconic images for NTD in the countryside of Brazil in the decades of 1930–1950, such as “Washerwoman” (Fig 4A), the “Retreatants” (Fig 4B), showing a poor family coming from Northeast Brazil to a larger city searching for better working and living opportunities, (iii) scenes of death (Fig 4D and 4E), and (iv) scenes of children playing together (Fig 4H) or alone (Fig 4I and Fig 4J). In two of these paintings Portinari portrayed children showing CD Romaña´s sign (Fig 4C and Fig 4G). A detailed description of the activities held at all the wagons will be published elsewhere.

The general structure of the CE21 educational modules dialogues with the main objectives of each space which were: (a) *Focus on the patient*: to increase visibility to the confrontation of CD and to the persons who demand diagnosis, treatment and care, advocating for the health rights of chronic affected persons; (b) *Support for affected persons associations*: to promote health by empowering people in areas historically affected by this endemic disease, and to encourage the organization of new associations; (c) *Innovation bringing hope*: spreading advances in science that allow solid health education for the prevention and treatment of acute and especially chronic CD; (d) *Demystification of the disease*: to inform people of endemic area and health professionals that the infection is not synonyms of disease, since seven out of ten persons seropositive for *T*. *cruzi* infection do not develop the disease. During the acute and the chronic phases, the seropositive person can treat the infection with antiparasitic compounds, and during the chronic phase, relevant signs may be mitigated by other symptomatic treatment.

The activities of the Expresso de Chagas XXI (Table 1, Figs 2 and 3) focused on the main scientific concepts that would be important to present and experience by the participants. We also focused on artistic strategies that could contribute to expanding the participants’ imaginations as proposed by other authors [26–28]. The contents seek to disclose that: (i) there are measures to be taken to live healthy at any stage in which the infection or disease is discovered; (ii) infection can be prevented with information and knowledge, and can be treated with antiparasitic drugs; (iii) follow-up of infected people in primary healthcare units is very important; (iv) diagnosis is available free of charge in the public health system in Brazil and the medical doctors are responsible for ordering diagnostic tests; (v) the medicine is freely supplied by the Brazilian National Health System; (vi) rehabilitation programs for those who present heart or digestive disorders are available; (vii) there are Associations of affected persons that empower and congregate all the people interested in facing this problematic. Documents related to the research projects and to logistic initiatives assumed previously to the implementation of the expedition are available at the public platforms reported in Methods section (*vide supra*).

### Adherence, interest, and profile of citizens attending Chagas Express 21 activities

The activities started during winter vacations in Brazil, at the northeast of Minas Gerais, an important endemic area for CD in Brazil [11]. The crowded entrance of people invited to participate in the activities (Fig 2F and Table 2) was a sign of high adherence/concern. We exceeded our own expectations and the CE21 team needed to increase the staff at the entry and registration station (Fig 2C–2E). A significant number of interested people came to participate on the project (Fig 2F and Table 2) entering the scenography by the Station “Lassance” (Fig 2B).

**Table 2:** Chagas Express XXI quantitative indicators and results: profile of participants, knowledge about Chagas disease, engagement, and awareness.

INDICATOR	Total	GM	Esp	MOC	Las	BH
Total days of activities	9	2	2	2	1/2	2
**Sociodemographic profile**						
Number of participants filling the identification forms	2,117	414	1,145	352	76	130
Female (%)	62	61	62	54	65	72
Male (%)	38	39	38	46	35	28
Age 1–15 (%)	17	27	15	6	37	2
Age 16–30 (%)	18	15	13	25	14	63
Age 31–45 (%)	24	21	24	40	21	21
Age 46–60 (%)	25	20	29	26	17	10
Age 60–95 (%)	16	16	19	4	12	4
No education (%)–E1	8	11	12	0	7	0
Fundam. Education (%)–E2	45	56	59	12	42	3
High School education (%)–E3	19	22	19	49	29	11
University education (%)–E4	11	4	5	30	15	67
Specialists (%)–E5	2	2	1	8	3	16
Lower education level (E1+E2) %	59	70	75	22	48	3
Higher education level (E3+E4+E5) (%)	41	30	25	78	52	97
Did not informed (%)	3	5	4	1	3	3
**Chagas Disease knowledge**						
Number of answers (n)	1908	414	1144	142	78	130
Yes, I have heard about CD (%)	85	78	88	97	76	98
No, I never heard about CD (%)	15	22	12	3	24	2
Yes, someone in my family has/had CD (%)	33	46	34	30	22	25
I don´t know how we get CD (%)	34	41	35	8	51	5
No, I don´t know how to treat CD (%)	81	86	86	¨64	87	42
**Chagas disease knowledge according to education level**						
Total numbers of E1+E2 answers (n)	1214	276	816	81	37	4
No, I never heard about CD (%)	19.2*	28.2*	14.7*	22.2*	40.5*	nd
Yes, someone in my family has/had CD (%)	42.7	52.5	41.0	37.0*	21.6	nd
I don´t know how we get CD (%)	43.5*	47.5*	40.8*	44.4*	70.2*	nd
I don´t know how to treat CD (%)	88.4*	88.4*	89.9*	75.3*	83.8*	nd
Total numbers of E3+E4+E5 answers (n)	833	115	271	286	39	122
No, I never heard about CD (%)	1.8	4.3	2.9	0.7	0	0
Yes, someone in my family has/had CD (%)	35.4	52.2	38.7	29.8	35.8	1.6
I don´t know how we get CD (%)	12.5	17.4	18.8	8.7	12.8	2.4
I don´t know how to treat CD (%)	64.2	76.5	71.5	60.8	76.9	40.2
**Engagement and consciousness**						
Contacts for Health Promotion Nuclei / Chagas disease Associations	629	145	339	130	15	Nd
Contacts for WhatsApp Groups	622	139	329	139	15	Nd
Embryos of CD Associations	5	2	1	1	1	Nd
Stakeholders contacted	89	9	24	39	5	12
Meetings with health professionals	10	2	4	1	1	2
Inter-sector meetings	1	0	1	0	0	nd

Cities: GM = Grão Mogol, Esp = Espinosa, MOC = Montes Claros, Las = Lassance, BH = Belo Horizonte. nd = not done.

* indicates significant frequency differences between lower and higher educational levels of the population (Chi-square test, p<0.01).

After the participant’s entry at Station “Lassance” the attending personnel distributed a badge with a lining (Fig 2E and 2I) indicating the six wagons. The participant was expected to fill the empty spots tagging the wagons that he/she had experienced. When the visit was completed and all the tags on the badge back were fixed (Fig 2I), the participant was considered ready to receive a participation certificate (Fig 2J) labeled with the official stamp of the Oswaldo Cruz Institute, the same research center where Carlos Chagas worked and directed. In the third day of the expedition the first 1,000 certificates previously printed ran out. At the end of the expedition, 2,000 certificates were awarded. As referred orally by many participants, this was frequently the unique certificate he/she received during his/her lifetime (Fig 2J), thus configuring another empowerment strategy.

During the nine days of expedition, CE21 engaged 2,117 people (GM 464 / Esp 1142 / MOC 352 / Las 76 / BH 139), as shown in Table 2. This important number of people exceed our expectations since a previous attractive event (https://webterra.com.br/2019/02/26/1a-edicao-do-projeto-educacao-em-saude-e-realizado-em-espinosa-e-sao-francisco/) did not reach 200 persons, as reported by our colleague and co-author (TMV). Accordingly, we estimated 500–1,000 participants accepted at the ethical committee.

According to gender, most of the participants (62%) were female (Table 2). A higher percentage of men (46%) was noted in MOC, where many male agents for endemic control registered presence and actively took part of the ArtScience activities. Age 31–60 years old participants were predominant (49%), with the two extremes of the age range accounting for 16–17%. A higher percentage of children taking part of the ArtScience activities was observed in Lassance, where the exhibition stayed for only half of a day (Table 2). This explained the relatively low number of participants (n = 76); local media information attracted families to visit the exhibition. On the other cities, even aided by the local media, municipality health services motivated focal and specific people to visit CE21.

The answers related to education (Table 2) elucidated that most participants attained only complete or uncomplete elementary school grade, especially in the small country cities (e.g., 70% in GM, 75% in Esp). In MOC, the largest city of the endemic region 78% of the public bears a higher education level, as well as in BH (97%, as expected for the attending persons of a university congress). Concerning their basic knowledge related to CD, 85% of the participants have already heard about CD and 33% reported that someone in the family has/had CD. However, three impressive results indicated that in that endemic area nearly 15% had never heard of CD, 34% ignored how CD is transmitted, and 81% did not know how to treat CD. We compared the responses of the participants according to their level of education: (E1+E2) corresponding to persons with no education or just the fundamental schooling level, and (E3+E4+E5) corresponding to high school and university levels. Despite the similarity in the frequencies of both groups regarding the family experience of coping with CD in the family, the frequencies of the responses regarding having heard about CD and knowing about transmission and treatment were statistically different between the participants of the two groups (E1 + E2) x (E3 + E4 + E5), p <0.01) in the four endemic cities visited (Table 2). The higher differences were identified in the first two questions. The question related to treatment showed a 75 to 90% lack of knowledge among the participants with the lowest level of education. Moreover, 60–76% of people with a higher education level showed unawareness thus emphasizing the urgent need for health education and CD in this region. An astonishing result was observed in Belo Horizonte, where 97% of the participants had high school / higher education and, presumably, were interested in Tropical Medicine since they were participating in the Brazilian Congress of the Society of Tropical Medicine. Of these, 40% were unaware of CD treatment. Although we did not apply a knowledge survey conducted before and after the EC21 activities, oral testimonies indicated their surprise in getting to know the possibilities of treating CD in different phases. One worrisome result was the frequent lack of knowledge of health professionals concerning the availability of treatment guidelines [10] published in 2018 but remained unknown by mid-2019 in those endemic areas.

### Organizing civil society and fostering citizenship: community engagement and self-consciousness

Another important result was the registration of over 600 participants interested in collaborating and/or participating in civil organizations of health forum, such as the proposed “Nuclei of health promotion” (n = 629, Table 2) and/or new local “Chagas disease Associations” (n = 622, Table 2). These persons were invited to join a “WhatsApp” group to receive information directly from Rio Chagas Association members and CE21 experts, and to share ideas and proposals for new local activities, as well as new requests from local citizens concerning their needs related to CD diagnosis and/or treatment. The idea of organizing the “Nuclei of health promotion” intended to extend their activities beyond CD and to account for the proposal of *One Health education strategies*, integrating different vector-borne prevalent infection diseases in the region, and to focus on health promotion, well-being, and quality of life, and not only to a specific disease. These ideas articulate CE21 strategy with the United Nations Sustainable Development Goals (www.un.org/sustainabledevelopment/). In each city visited by the CE21 Rio Chagas Association a new association “was seeded” (Table 2) and an old one (that was inactive for decades) was revitalized. The city of “Fruta de Leite” did not receive any specific activity from CE21 but encouraged its health professionals to visit CE21 in the neighboring cities of GM and MOC. An image of one of these meetings in shown in Fig 3H, where the centrally located woman is a community leader holding a set of documents given by the members of Rio Chagas Association to help organizing a local Association. After almost six months of the Expedition, two Associations were organized, with meeting agendas and activities planned each year for the World Chagas Disease Day, April 14^th^.

### Engaging stakeholders, local health professionals and media

To explain any subject to the people is a matter of decision of the person, group, organization, or institution that intends to communicate and does not necessarily need the engagement of governmental agents´. However, when dealing with CD issues, or any other health-related subject that is a consequence of socioeconomic determinants, the communicator must keep contact with the health and education authorities, as well as the commitment, given the great expectation that can arise after the increase of awareness and knowledge of a vulnerable population. To implement CE21 all the municipality majors and their main health stakeholders were contacted prior to the expedition, as well as the health planning and surveillance sector of the Minas Gerais state. This strategy was a fundamental step both to link the CE21’s non-formal education proposal to schools and universities in the host cities, as well as to compromise the local health system in managing the serological results of the participants with a positive test for CD. The total number of stakeholders that were contacted during CE21 activities as well as the number of meetings is shown in Table 2. The meetings, held directly in the field, provided an opportunity for discussion with specialists in CD as well as the knowledge of the new treatment guidelines [10] that remained unknown to most agents and health professionals. Fig 3G shows one of those meetings that took place in the city of Espinosa. These results, *per se*, are a great challenge for the health system when providing integral care for these affected and positive persons. CE21results also made it easier for people organized in social movements to pressure and defend actions and policies to access their rights, now recognized in the new Brazilian guidelines for the treatment of CD [10]. To manage this challenge, local authorities must partner with these initiatives. Concerning communication strategies, virtual communities were created to interact with the participants in different ways: Facebook, to post articles and reports on the disease; Instagram, to publish daily stories of CE21 full coverage (live and general information); YouTube, to publish videos derived from CE21 activities and about CD. About 600 people followed the development of activities on social media, mainly on Instagram and Facebook, a number considered relevant. The media impact was perceived from journalistic reports in print newspapers, TV, radio, and local, national, and international websites. Before the expedition, the disclosure of the project started in local TV and radio shows. During the expedition CE21 was on the cover of the “Gazeta”, an important printed newspaper in the northeast of Minas Gerais. More than 20 websites published reports praising and signaling the importance of similar projects to CE21 in this endemic region, and even internationally (www.coalicionchagas.org/en/news-article/-/asset_publisher/hJnt8AyJM2Af/content/expresso-chagas-21). Taken together, all these actions brought visibility to issues regarding CD and a greater engagement of interested people.

### Participants´ perceptions of Chagas Express XXI ArtScience activities

**Fig 5:**
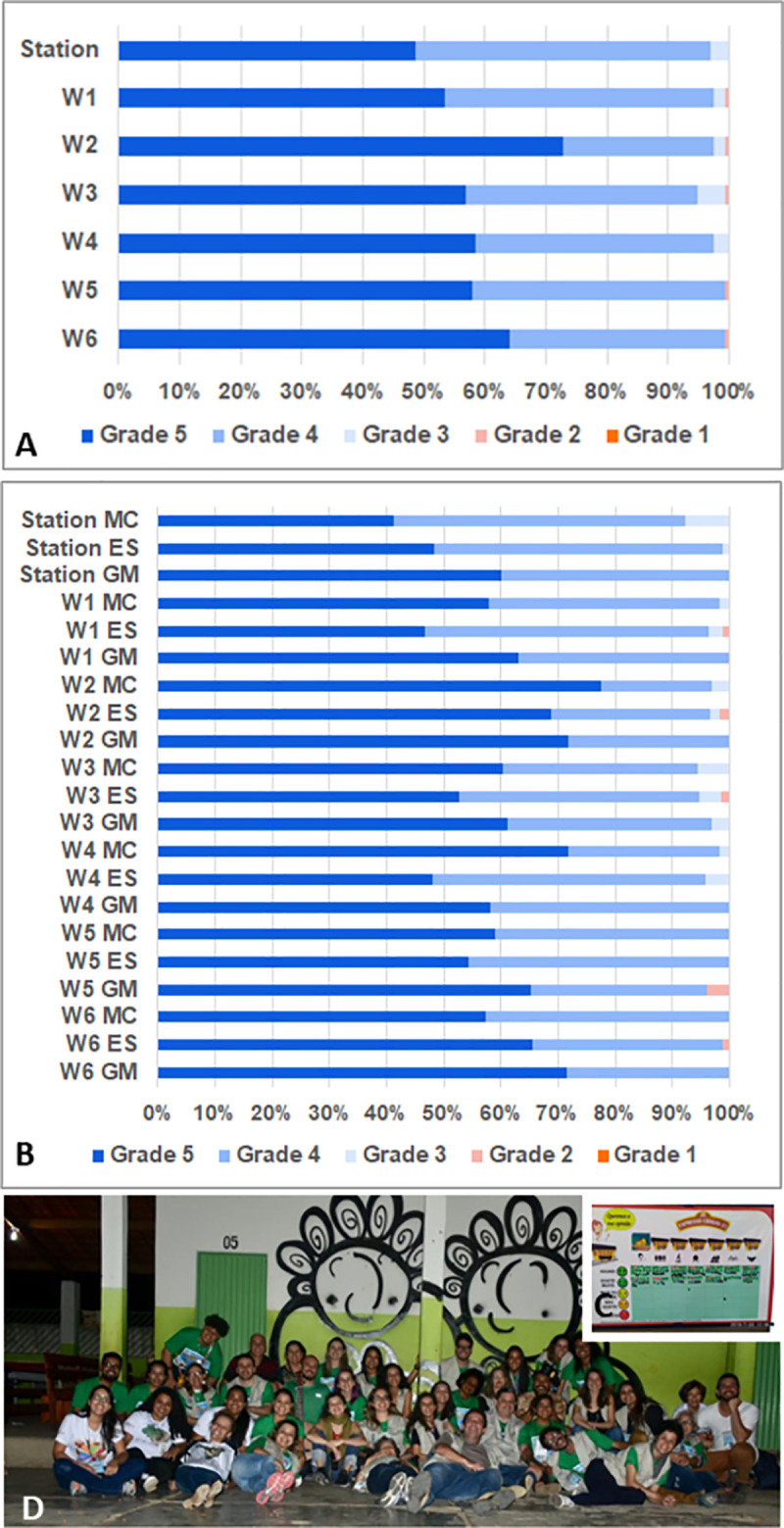
**Chagas Express public evaluation and mural painting legacy**: (A) Global evaluation of each space of the exposition expressed as the percentage of stickers inserted in each wagon frame. “Station” corresponds to entry/exit module and “W1” to “W6” corresponds to wagon 1 to wagon 6. Only the participants attending the activity of wagon 6 participated in the evaluation study and the total number (n) of bullets (100%) were of 171 in the Station Lassance, 163 for wagon 1, 155 for wagon 2, 158 for wagon 3, 164 for wagon 4, 133 for wagon 5 and 186 for wagon 6. Grade/Points were expressed as emoticons: Grade 5 = I love it; Grade 4 = I liked it very much; Grade 3 = I liked it more or less; Grade 2 = I didn´t like it; Grade 1 = I hated it. (B) Similar evaluation of Fig 5A, shows the specific data collected at the three different cities visited by Chagas Express XXI, as to know: MC = Montes Claros, ES = Espinosa, GM = Grão Mogol; (C) Thumbnail image of the evaluation chart: participants received a set of seven adhesive bullets and were asked to stick them at the frame square corresponding the attributed grade to each wagon and station. (D) Mural graffiti painting in the school yard at Espinosa.

Since the non-formal teaching-learning process occurs as multiple strategies, activities, and mind tools, the participants were invited to evaluate their experience on the Chagas Express train, using a Likert scale with 5 points (Fig 5A–5C). After completing the proposed set of activities, but not necessarily participating in all of them, the participants received in wagon 6 a set of seven sticker tags. In the absence of the mediator, they were asked to fix the stickers at the frame square (Fig 5C) corresponding to the grade they would attribute to the activities developed in each wagon and station. Emoticon faces indicated the 5 grades: “I love it” /5; “I liked it very much” /4; “I liked it more or less” /3; “I didn´t like it” /2; “I hated it” /1 (Fig 5A and 5B). The CE21 psychologists mediated the evaluation and photographed the chart at the end of each day (Fig 5C). Data were collected and processed as the number of tags in each grade of the scale. The total number of sticker tags for each space were determined and the corresponding percentage was calculated for the total number of persons that evaluated each space of the exhibition daily (Fig 5A). Results from 2 days in the same city were summed and the evaluations of the same spaces at different cities were plotted aside (Fig 5B).

At least 95% of the evaluators loved or liked very much the activities (Fig 5A–5C) in all spaces, and over 50% loved (dark blue) the activities of all the 6 wagons (Fig 5A). The wagon 2 (innovations/ microscopes/ immunefluorart) was the best evaluated educational module. When the performance of the exhibition was compared in the three cities evaluated (Fig 5B), interesting and subtle differences were noted. Wagon 4 (One Health/vectors, reservoirs, environmental diversity) was better evaluated in MOC, a city where many service workers of health surveillance teams participated intensively. Station Lassance and wagon 6 were better evaluated in GM, where the stress for the waiting in line was lower with better interaction between the crew of mediators and the public, which probably explains the result shown in Fig 5B.

### Qualitative data indicated art as a seminal strategy and a leftover

CE21 gathered various experiences and intensified interpersonal and intersectoral relationships of health professionals, academics, researchers, and local populations. In wagon 6, at the end of their experience, we collected perceptions of participants by freely recording their testimonials, with their specific agreement to have them published at the CE21 YouTube channel. Here we selected the best moments of some attendant’s speeches and presented transcriptions to build a general perception of the participant’s feelings about the CE21 experience.

Among health professionals, the highlights of CE21 exhibition were knowledge on (i) *T*. *cruzi* cycle; (ii) Chagas disease vectors and (iii) wild mammals or oral transmission. An Endemic Disease Control Agent explained the importance of his role on CD mitigation in rural areas and reinforced the need of continuous education about prevention actions and CD treatment. “… *knowledge is wealth*!” he stated. A community agent sang in an emotional freestyle: “*I am extremely happy*, *it was really good to be here*, *I am leaving here my endearment and hugs*, *I was very welcomed by all of you*, *thanks god*… *I love you all*!” These words were significantly touching for the CE21 crew. The network of collaborative attention brought well-being and turned CE21 into an amazing experience to participants. A university employee remarked: “…*I think that it was an incredible exhibition and the set up was quite simple*. *We are so comfortable and at the same time well-informed*…”. In Espinosa, a citizen reported another perception: “*We should clarify and move straight to dissemination of Chagas disease knowledge about the needed cure and to inform the population of this remarkable rural area*, *that will become an urban area*, *because of the deforestation*. *So*, *the kissing bugs*, *the marmosets and the other vectors are coming to town*. *It will no longer be a disease of the poor people*, *but a disease of us all* …”. These impressions corroborate the success of the purpose of the ArtScience projects to be carried out in those endemic regions.

Education professionals were also interested in joining CE21. A professor at Unimontes said: “… *the researcher does its work to contribute and improve people’s lives*, *especially the person who lives in endemic area that could face the disease*…”. She congratulated the joint efforts of professionals involved in CE21 that shared their knowledge with the community. She also suggested that the new CE21 approach should spread to higher education, such as for bachelor’s courses. Taken all participants perceptions together, we can suggest that the impact of CE21 experience was a real social legacy, an amazing experience that brought well-being and stimulated the participants for a more critical awareness about the CD complex issue.

Oral reports were collected not only from all the participants who agree to record voice or video briefs, but also from all the CE21 team members, who also produced written reports. Two not foreseen results were stressed in reports of the CE21 team: (1) the organization of an ArtScience research group to establish a closer cooperation process and facilitate exchange between Unimontes (www.unimontes.br) and Fiocruz (www.fiocruz.br), the two main CE21 implementing institutions, and (2) the muralism art on the walls of the cities. EJC, the main plastic artist in the CE21 group, collectively produced four mural paintings, one of which is shown in Fig 5D. These graffiti murals became a project mark in the schools that received CE21 and, mostly, they were demanded and supported (regarding to the cost of painting materials) by the schools´ boards.

### Active search of chronic Chagas disease cases and serology results

The second objective of CE21 was to test this social technology as a tool to actively search for cases of chronic asymptomatic CD. To comprise with this objective, a serological diagnosis of CD was offered in three municipalities (GM, Esp, and MOC) as part of the activities of wagon 2. People volunteered to collect blood and reference diagnosis centers tested the samples, using two different CD tests, as described in Methods section. As shown in Table 3, 56% of the participants asked for CD blood testing and even if the participant was interested only in getting a free diagnosis, he/she was encouraged not just to collect the sample, but also to participate in the activities offered. We offered the registration and we also explained the importance of passing through activities, at least, in wagons 1 and 2. Then, they obtained the password to diagnostic access and were informed that the local health professionals would contact them in 30–60 days to provide the results. In positive cases, it was clarified that the necessary and sequential health care for the clinical follow-up of CD, with all the laboratory complement, clinical exams and treatment options are guaranteed by the Brazilian guidelines formally presented in the PCDT [10].

**Table 3:** Serodiagnosis survey during Chagas Express XXI expeditions.

INDICATOR	Total	GM	Esp	MOC
Total days of activities	6	2	2	2
Blood samples (adults)	1,110	232	719	159
Samples positive for Chagas disease	222	56	133	33
% of positivity (adults)	20	23.7	18.5	20.7

We observed a high percentage of positivity: from the 1,100 adults tested, 20% were diagnosed as positive cases for CD (Table 3), varying from 18.5% in Espinosa, to 23% in Grão Mogol. Informing positive results was not an issue planned for the CE21 team to provide, but a responsibility of health representatives in each city. Follow-up of those positive cases in the local primary health units was committed by local stakeholders and the corresponding representatives of public health units of each city, as a direct consequence of the close interaction established since the planning step of CE21. A planned joint meeting with the research team and one health representative of each city that participated in the study and expeditions took place at Fiocruz (Rio de Janeiro) in January 2020. The evaluation meeting discussed some barriers and challenges to further etiological and symptomatic treatment, as well as healthcare for the positive cases. Oral reports of health managers confirmed that citizens “*empowered by CE21*” (in their words) were debating with local health services to request what was proposed in PCDT [10] and advocating for their civil rights for health care.

## Discussion

### The power of talking about Chagas disease with ArtScience: validation of Chagas Express XXI social technology and its availability for active search of asymptomatic chronic cases

The results obtained during CE21 expedition could support and validate (*vide infra*) the proposal that ArtScience is an excellent approach to introduce and to communicate science and health subjects to any segment of the public: health and culture professionals, scholars, teachers, children, adults, seniors, of diverse education levels. At least four evidence support this conclusion: (1) ArtScience articulating with the relevant subject of CD attracted an impressive number (over 2,000) of people to the exhibition, not seen in those cities before, even when attracting events were performed. The difference from other communication calls was not the information nor the diagnosis components but the active and creative learning chances offered at CE21; (2) There was an intense impact of the ArtScience activities in engagement/ concernment, and satisfaction of invited people to the activities. We received many requests for meetings with the CD specialists of our team concerning treatment guidelines and this highlighted the need for permanent education and training of health professionals on CD issues in the endemic areas.; (3) Complex subjects could be easily communicated using ArtScience strategies and tools, as shown in the subject list of contents in CE21 (Table 1); (4) Approaching specific topics with ArtScience made it possible to achieve a huge number of satisfied participants, throughout the process. These conclusions emerged from the results presented here and fit the main publications on ArtScience concept and approach [17–28,44,45]. They validated the CE21 social technology as suitable to promote community engagement, as indicated by the numbers highlighted in Table 2, thus allowing us to be certain that the first objective of the work was accomplished.

The interest of health professionals in a non-formal and freely invited activity was high and sustained many specific meetings, attaining dozens of professionals who posed questions and asked for more opportunities of education. Health professionals must be updated based on credible sources, promoting the improvement of their knowledge [46]. In Colombia, another endemic country, the lack of awareness of the disease was observed using a questionnaire to evaluate the knowledge among physicians concerning recommendations on the diagnosis of CD [47]. Untrained and not updated MD attending chronic affected persons may not request CD diagnostic tests, as we heard from some participants in our field expeditions. Thus, initiatives such as CE21, composed of students and CD experts that dialogue both with citizens and with health professionals, are important in endemic areas.

The education/communication approach was shown to be a valuable tool for healthcare providers [48] but also among the people residing in an endemic area, which may be able to recognize the endemic vector [49]. In addition, the present approach was instrumental in promoting the community awareness and it is relevant among patients [50], presumably encouraging diagnosis and treatment. In this regard, the education/communication activities were associated with the disease surveillance in the present study as the public was able to perform CD diagnostic. The conventional education is generally not related to the student environment, so is seldom attractive and does not necessarily uses reality as an anchor for problematization and questioning [16]. Much like the Paulo Freire method, the CE21 focuses on a local epidemiologically important neglected disease. Furthermore, the ArtScience approach shows the theme in a clear form, attractive even to adults that left school during childhood, a special target population in CD endemic areas.

CE21 results also demonstrated that ArtScience approach strengthens the links and build bridges among people interested in the same subject. These outcomes led to very productive participation in the workshops concerning different subject scenarios integrated in the full exhibition, as well as to positive evaluations and meaningful expressions of people after completing the exhibition circuit, as shown in Fig 5. The ArtScience approach was formally reported by Siler [26,27]. He hints to stress associations among thoughts and analogies, to foster imagination, creativity and innovation following an imaginary stair with the successive steps of imagine, connect, discover, invent, apply, and innovate. We associated this approach with the 13 creativity tools proposed by Root-Bernstein and Root-Bernstein [28]. The coupling of these two approaches led us to adopt the concept of ArtScience to introduce transdisciplinary practices and theory [19,24,25] and to produce over 40 activities that boosted the imagination and perception skills of the participants.

With the CE21 validation concerning local mobilization, interest, and engagement, the second objective of the work was also confirmed: CE21 may be a suitable tool to perform the active search of chronic asymptomatic CD cases, to better organize integral care for those affected. However, the greater interest in blood screening, more than in carrying out educational activities, was probably due to the lack of diagnosis opportunities in such remote endemic areas. CE21 potentiated this demand by offering a unique, innovative, and unexpected cultural event that was thematic centered in CD. The public health relevance of the findings of 20% seropositive (asymptomatic) carriers and unawareness of the treatment options is a matter of important concern to both CE21 researchers and local stakeholders. It means that effective disease control measures are urgently required in those cities, articulating, and integrating CD prevention through vector surveillance and community education with health promotion strategies through civil organization and dialogic circles at primary health units. Evidently, those actions depend directly on political decisions and on social pressure to face the negligence concerning CD. The expectations were that both the creation of nuclei for health promotion and the CD new associations could induce local governments to implement and sustain CD integral care. To strengthen the affected population engagement in those endemic areas, we intentionally used the term “Chagas disease affected persons”, instead of “Chagas disease patients” or “chagasic patients”. “Patients” refers to a concept that "reduces" or limits them to their condition of "people with a health problem" only defined from their link with the health system, an image that ultimately contradicts the emancipatory proposal of the experience reported. In this context, Freire´s pedagogy was especially helpful, since the Rio Chagas members emerged “from” the people, engaged with artists and with scientists and all together, we talked “with” the people and not “for” the people.

### Lessons learned during Chagas Express XXI

Many lessons were learned after such an intense experience of interpersonal encounters assembling a team of more than 60 researchers and educators and a population of more than 2,000 interested people.

**Fig 6:**
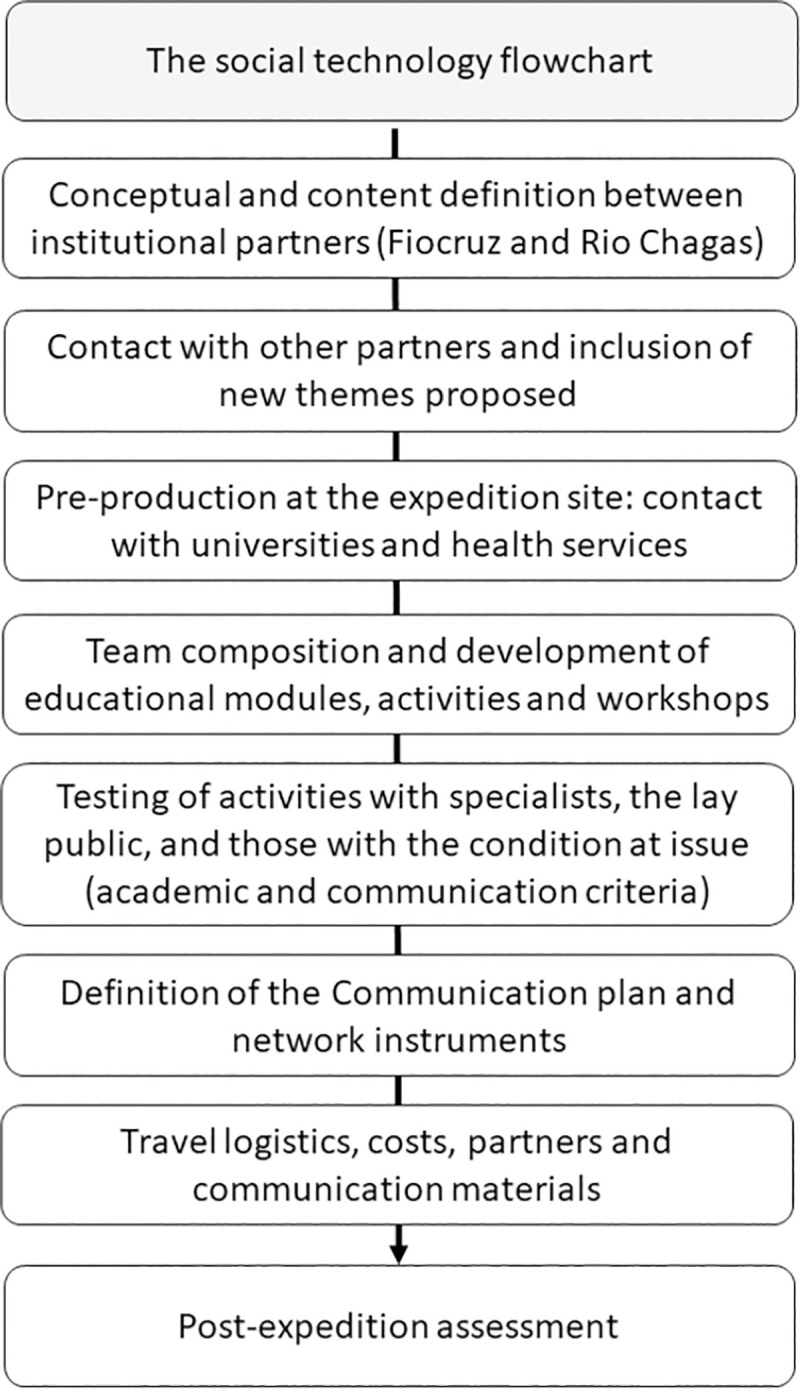
Flowchart of Chagas Express XXI educational social technology: an eight-step process.

1. In an especially important endemic area for CD surprising data strongly suggest that further activities are needed to decrease the misinformation about a such relevant subject: 81% of the participants did not know that treatments are available, 34% did not know how the disease is transmitted and the risks of infection, and 15% never heard about the disease, thus stressing the need for new information policies for all, and especially to people with the lower educational level, an important determinant of vulnerability.
2. The public is avid for scientific and health information and knowledge sharing; correct messages are missing in these (and probably also in other) endemic areas of Brazil. They searched for the information whenever possible (as CE21 offered them) and answered questions, interacting pleasantly with the academic group. A limitation of the study was the absence of pre/post testing to measure knowledge gain after the participation. Even though we prepared forms to assess knowledge, attitudes, and practices (KAP) for this approach, the high number of attendees to the exhibition oriented the repositioning of CE21 team members. Thus, we choose to focus on the general knowledge, on the blood collection and on the diversity of ArtScience activities in all CE21 presentations, giving up this specific KAP study for future opportunities. The collection of testimonies in wagon 6 partially accomplished for this gap and allowed us to assume the general satisfaction of the public with the proposed activities, expressed by 95% satisfaction using the Likert scale.
3. Health professionals, specifically endemic and community health agents (that were more frequent in MOC as CE21 participants), need access to capacity building courses to decrease unawareness about biological and epidemiological concepts related to CD, focusing on the One Health approach [43].
4. CE21 is a potent tool to implement active search of chronic cases of CD in endemic areas. CD is an almost forgotten or “invisible” disease, since 80% of the seropositive cases are asymptomatic according to the last Brazilian national survey for adults, dating from the eighties [51]. Even though the high interest of the population in getting access to blood testing for CD diagnosis cannot be directly attributed to the education activities *per se*, the intensity of the social impact of ArtScience CD activities attracted enough attention to transform a cultural and health promotion event into a special day to talk about and to diagnosis CD. This is the essence of active search: to find the vulnerable people and to offer them the conditions to overcome a specific problem, in this case the lack of knowledge about his/her specific CD status, as a person exposed to risk-of-transmission conditions. Based on epidemiological data [11] and after the important advocacy of FINDECHAGAS, Médicins Sans Frontières and scientific society [6], the Brazilian government finally acknowledged that chronic cases will be of compulsory notification [11] but there is not yet any specific action or tool for active search of such cases. CE21 showed up to be useful to detect them. The finding of 20% of positivity in the CD tested samples confirms the high prevalence of chronic cases in those cities. However, one limitation of the study is that the 18.5 to 23.7% of seropositivity found in CE21 data could not be taken as the actual cities´ infection rates, since it does not represent a prevalence designed study and was biased by the interested population to get access to diagnosis and information. Precise chronic prevalence data are still lacking but may reach 2.4% of the Brazilian population [8]. The recent technical report of the Brazilian Ministry of Health [11] states that: “*The challenges are still enormous for facing CD*, *this silent illness that*, *in addition to the burden of morbidity*, *mortality*, *disability and stigma*, *imposes a social and relevant social burden financially relevant*, *mainly on groups economically less privileged and marginalized”*.
5. CE21 empowered the local population to fight for the rights to access health care and health knowledge [52], as shown by the important records of participants´ perceptions in their testimonial videos. As stated by Barry [52], “*rather than compassion for inequalities*, *vulnerabilities*, *deprivations and misery*, *or bad fate*, *foci such as social justice*, *preparedness*, *and empowerment are of utmost importance*” as the contribution of social sciences studies and practices dealing with neglected diseases. CE21 built knowledge with local CD affected people, seeding groups of interested people for the organization of new local CD Associations, according to the pedagogy of autonomy studied and proposed by Paulo Freire [16].
6. CE21 is a strong tool for students´ learning, especially during the MSc and PhD science education process: they composed *circa* 70% of the expedition team and learned on field activity what could only be imagined in theoretic classes and read in articles. Many of them were not related to CD projects and reported that the expedition was a unique educational and, moreover, personal experience.
7. CE21 can be considered as a social technology, following the concept developed by the Brazilian Institute of Social Technology (www.itsbrasil.org.br) and reexamined by academy authors [38,39]. The following characteristics allow CE21 categorization as a social technology: (i) it was built upon a partnership between academy and society, (ii) following a social request, and (iii) could be appropriated/ adapted / transformed by the community itself (iv) generated and aggregated new tangible or intangible values to the local and directly affected people. As an intangible value, in this case, we are discussing about health information and care, including selfcare. A flowchart summarizing the social educational technology developed by CE21 is presented in Fig 6: eight steps need to be conceived, prepared, and implemented. The inability of dealing with any of those steps would represent obstacles to reproduce and to expand the technology. The possibility of composing a clear chronic CD picture in each municipality and then structure operational activities related to CD management is a great opportunity that CE21 brings to local leaders the intent to face this public health issue. It would be a mistake to consider that these objectives are directly interdependent, since increasing awareness/ knowledge/engagement and increasing the mapping of chronic CD prevalence through active search of asymptomatic cases are two interconnected goals but are not precisely a cause and a consequence. We consider that CE21 may be appropriate to other endemic cities and it is possible to adapt it to different CD and climate scenarios. However, at least one limitation needs to be stressed: this is a case study carried out in a specific region of Brazil; with a specific and motivated team, engaging with CD affected person´s association. Its reproducibility is not a goal that could be directly achieved. Testing this social technology in other places and with other groups of engaged people would help to confirm if it can be fully adapted to a new situation, as we suppose and encourage.

### Chagas disease affected persons as main actors in Chagas Express XXI

CD affected persons, students, researchers, and a variety of health professionals are the different actors involved with CD issues. As a transdisciplinary product, CE21 highlighted those protagonists either by offering ArtScience activities and by stimulating community engagement. The success obtained by this technology in Brazil was due to this dialogical perspective, following Paulo Freire´s pedagogy [16]. We believe that the most important differential in this technology was the participation of CD affected persons: “*Nothing about us without us*”, following the mental illness rights movement mantra [53,54]. Members from Rio Chagas Association pushed the first ideas of traveling to their homeland areas in 2018 and were co-creators, co-evaluators and co-participants of all the CE21 embedded activities in the 2019 expedition. Local stakeholders and members from the local Health System also helped to identify the best cities, to propose the expedition roadmap, and to get all the help to set up blood sample processing and testing. The rich experience of CE21 encourage other projects to benefit from inviting affected people and local health services and asking for their opinion on what is important for researching, from preparing the study agenda to conducting research itself, to deconstruct the single view of patients and affected people as “subjects” of the studies and to venture joining them as protagonists in research projects.

CE21 completely changed the regular role of “patients” in research studies thus influencing the course of the research. The passive role of the subjects was also rejected in a previous work [55], leading to patients´ influence over a research that could affect their lives. With their own expertise, they contributed from the initial conception of the project, helped to choose Minas Gerais cities and changed project identification from CE XXI to CE21. Low level of education is a general profile of CD seropositive individuals [56–58], as observed in the present study. Most CD affected persons have a low income, and restricted access to education opportunities. Changing “XXI” by “21” helped to simplify the understanding of individuals in the endemic area to CD.

Another important feature observed in CE21 experience was the full engagement of Rio Chagas Association members, persons in different stages of the infection and the disease, and other family persons or friends, referred as “affected people” [55,59]. ArtScience approaches makes it comfortable for CD affected persons to act as informal educators/ mediators of activities to sensitize local people, thus yielding the seeding of new CD affected person´s Associations and the compromise of hundreds to future popular Nuclei of Health Promotion. This was the most important legacy of CE21, a social legacy. Until CE21 expedition, the Brazilian Associations of CD affected persons were settled in São Paulo, Pernambuco, and Rio de Janeiro states. In fact, CE21 created embryos of four new Associations, an extremely relevant fact. Six months later of the meeting with health managers from the visited cities, we received information that two of them were already operating and urging the health secretaries to answer their needs. Besides, we also received the news that two other Associations were in process of creation in Goiás and Bahia states, another indicator that the affected population is increasingly motivated to self-organize into more active and effective actors.

## Conclusion

The two most important conclusions of the present work with CE21 are: (i) ArtScience is a powerful strategy to face complex health problems by increasing engagement in discussion of subjects and (ii) active search mediated by this social technology is a useful strategy to map out chronic asymptomatic cases of a chronic long-lasting disease. Sensitizing interested people to advances of science, innovations, and historical landmarks of a public health problem that involves One Health principles [43] help to build bridges among social actions, information needs, and biomedical/cultural issues. As Rubin wrote, “*art can bring out the best in science*” [60]. CE21 was a case of sharing knowledge and perceptions among affected people, their families, scientists, educators, and health professionals. As a natural consequence, populations could be empowered by the commitment of these actors to increasing experiences with science, art, and citizenship. A promising input to innovation appears when the academy (Fiocruz, universities, and local Research Institutes) and the health services (local, municipal, regional, and state) integrate through the transdisciplinary nature of ArtScience approaches and practices. This is critical to face the reemerging problem about the public understanding of science and its benefits, thus generating a true approximation between science and society in their real needs.
